# Optimum land cover products for use in a *Glossina-morsitans *habitat model of Kenya

**DOI:** 10.1186/1476-072X-8-39

**Published:** 2009-06-29

**Authors:** Mark H DeVisser, Joseph P Messina

**Affiliations:** 1 Department of Geography and Center for Global Change and Earth Observations, Michigan State University, East Lansing, MI, USA

## Abstract

**Background:**

Tsetse flies are the primary vector for African trypanosomiasis, a disease that affects both humans and livestock across the continent of Africa. In 1973 tsetse flies were estimated to inhabit 22% of Kenya; by 1996 that number had risen to roughly 34%. Efforts to control the disease were hampered by a lack of information and costs associated with the identification of infested areas. Given changing spatial and demographic factors, a model that can predict suitable tsetse fly habitat based on land cover and climate change is critical to efforts aimed at controlling the disease. In this paper we present a generalizable method, using a modified Mapcurves goodness of fit test, to evaluate the existing publicly available land cover products to determine which products perform the best at identifying suitable tsetse fly land cover.

**Results:**

For single date applications, Africover was determined to be the best land use land cover (LULC) product for tsetse modeling. However, for changing habitats, whether climatically or anthropogenically forced, the IGBP DISCover and MODIS type 1 products where determined to be most practical.

**Conclusion:**

The method can be used to differentiate between various LULC products and be applied to any such research when there is a known relationship between a species and land cover.

## Background

### Introduction

African trypanosomiasis is a parasitic disease transmitted by the tsetse fly (genus *Glossina*) to animals and humans. It is a neglected tropical disease [1,2] and considered one of the most important economically debilitating diseases in Sub-Saharan Africa [Oloo F: Literature survey on unpublished records on environmental and socio-economic impacts assessment on tsetse and trypanosomiasis interventions in Kenya. 2006. unpublished]. Three major epidemics have occurred in the past hundred years, one between 1896 and 1906, and the other two in 1920 and 1970 [3]. In 1986, approximately 70 million people were estimated to be at risk of exposure to tsetse [3]. A decade later, it was estimated that at least 300,000 cases of Human African Trypanosomiasis (HAT), commonly known as sleeping sickness, were underreported due to lack of surveillance capabilities, diagnostic expertise, and health care access [3,4]. In 2001 as a response to these limitations, the World Health Organization (WHO), with public and private partnerships, initiated a new surveillance and elimination program [3], during which approximately 25,000 new cases were reported annually [5]. Furthermore, in some areas, HAT symptoms were misdiagnosed as malaria, and therefore masked the overall number of new HAT cases [4,6]. Animal African trypanosomiasis (AAT), commonly known as nagana, also indirectly affects the lives of people in Sub-Saharan Africa because it can decimate livestock thus impacting nutrition and livelihoods. It is estimated that livestock productivity decreases by 20% to 40% in tsetse infested areas [7,8]. In Kenya where livestock production accounts for approximately 12% of Gross Domestic Product (GDP) [9,10], the economic burden of sleeping sickness is felt at both local and national scales [11].

**Figure 1:**
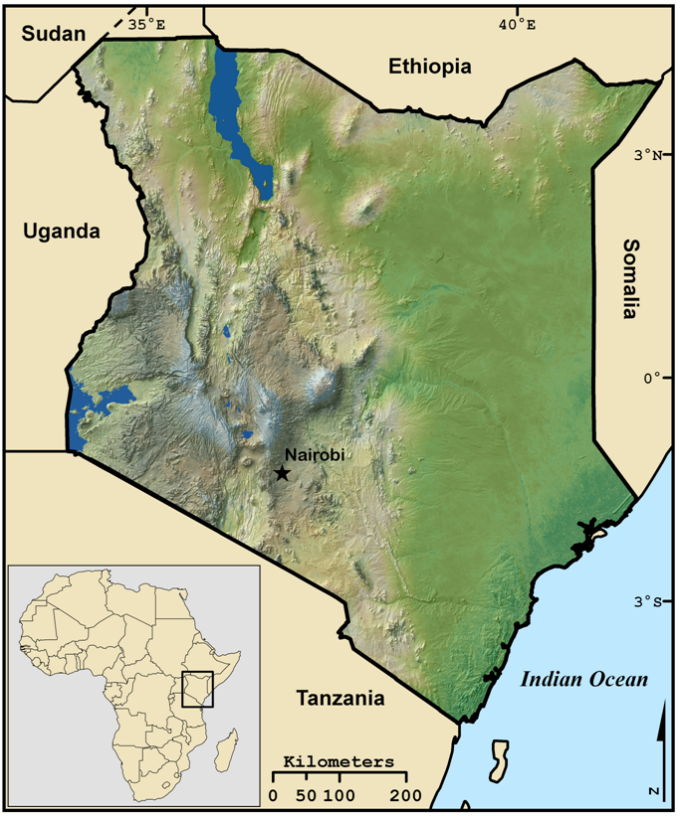
**Location and topography of Kenya**.

The geographic distribution of the tsetse fly varies throughout Sub-Saharan Africa and is closely linked to land cover [12]. Tsetse flies require land covers that contain vegetation greater than 3 cm in diameter and 1 to 4 meters in height, hereafter referred to as woody vegetation [13]. Habitats with suitable land cover range from the tropical rain forest to semi-arid grass savannah and wet mangrove, but in East Africa are specifically found in riparian and woody savannah ecosystems [14]. This study focuses on Kenya (Figure 1), where in 1973 tsetse flies were estimated to inhabit 22% of the country [15]. By 1996 the amount of Kenya estimated to be infested with tsetse flies had risen to roughly 34% [16].

Efforts to control the disease have been hampered by a lack of information and the substantial costs associated with the identification of infested areas, control traps, or broad eradication activities. Given changing LULC and climate factors, a model that can predict changes in suitable tsetse fly habitat is critical to efforts aimed at controlling the disease. Before constructing such a model the existing publicly available land cover products must be evaluated to determine which products perform the best at identifying suitable tsetse fly land cover. Rather than relying on reported accuracy assessments, not always available for each LULC product and expensive or impossible to perform post-production, we developed a generalizable method using a modified Mapcurves goodness of fit (GOF) test to identify the optimum land cover products. The method can be applied to any vector borne disease-modeling endeavor where a known environmental relationship between a given species and specific land covers exists.

### Tsetse flies and African trypanosomiasis in Kenya

Tsetse flies are divided into three sub-genus groups, all of which are found in Kenya. The sub-genus *Austenina*, also referred to as the *fusca *group, are commonly considered forest tsetse species, with the notable exception of *G. longipennis*, which lives in sparsely vegetated arid regions [17]. Three species within the *fusca *group are found in Kenya: *G. brevipalpis*, *G. fuscipleuris*, and *G. longipennis *[18]. The sub-genus *Nemorhina *or *palpalis *group, a riverine species group, with only one species, *G. Fuscipes*, is also present in Kenya [18]. The third sub-genus *Glossina *or *morsitans *group is considered a woody savannah tsetse species. Four species of the *morsitans *group are found within Kenya: *G. austeni*, *G. morsitans*, *G. swynnertoni*, and *G. pallidipes*.

**Figure 2:**
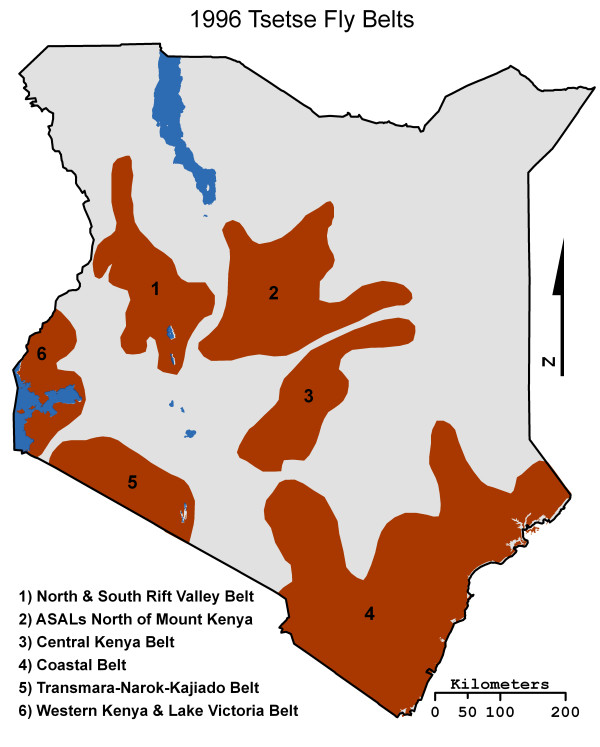
**The 1996 KETRI fly belts map**.

Although the eight species of tsetse fly in Kenya exist and live in diverse habitats, their populations are concentrated in six distinct zones: North and South Rift Valley, Arid and Semiarid Lands (ASALs) North of Mt. Kenya, Central Kenya, Coastal, Transmara-Narok-Kajiado, and the Western Kenya & Lake Victoria belts (Figure 2) [16,19]. The zones, commonly called fly belts, are infested with one or more tsetse species with boundaries set by a variety of physical, biological and anthropogenic barriers. *G. pallidipes *and G. *fuscipes *are the two most important tsetse species in Kenya because they are considered "efficient transmitters" of AAT and HAT. The tsetse fly vector carries the parasites to different animal hosts, allowing cyclical transmission, but the primary animal reservoirs are wild and domestic ungulates. Humans may also contribute to the reservoir pool [3], and both animals and humans contribute to trypanosoma genetic exchange [20]. In 2001 infection rates of cattle in select provinces of Kenya were as follows: Coastal, 15.6%, Rift Valley, 12.9%, and Western, 8.3% [9].

### Past modeling of tsetse fly habitat

Population density models characterized most early tsetse fly modeling endeavors and were primarily based on climate variables highly correlated with tsetse fly survival. These early models provided little in the form of predicted distributions. For example Nash [21] and later Bursell [22] identified humidity and temperature as key climatic variables influencing tsetse fly mortality, and both used linear regression to predict tsetse fly population densities. However, these models assumed that suitable habitat and tsetse flies were present at the modeled locations, and thus, in effect, only predicted tsetse fly population densities in known locations.

In 1971, Ford published what some consider the definitive book describing the ecology, history, control, and a variety of other topics concerning the tsetse fly across the African continent. Six years later Ford and Katondo [15] created the first widely accepted tsetse fly distribution maps based on field work and knowledge of the African landscape. Building on the work of Nash [21], Bursell [22], and Ford [14], the Trypanosomaiasis and Land-use in Africa (TALA) Research Group and the Environmental Research Group Oxford (ERGO), constructed several models dealing with tsetse flies starting in 1979 [23-27]. Initially the models were population density models [23], but later coarse resolution climate maps of temperature and vapor pressure were used to identify areas with suitable tsetse fly climate regimes [24].

Starting in the early 1990's remotely sensed land cover and climate data were employed to aid in identifying suitable tsetse fly habitat [28-30]. The Oxford group and the Programme against African Trypanosomiasis (PAAT) collaborated to create PAAT – Information System (PAATIS), a spatially explicit model that predicted tsetse fly distributions at a 5 km resolution using discriminant analysis and maximum likelihood statistics on remotely sensed environmental variables, socioeconomic data, and the Ford & Katondo [15] distribution maps [26]. The PAATIS model was later refined by ERGO, the Food and Agriculture Organization of the United Nations (FAO), and International Atomic Energy Agency (IAEA) employing logistic regression and produced 1 km spatial resolution predicted percent probability of particular tsetse fly species in various regions [27].

### Remotely sensed data and mapping vector-borne disease

Vector borne diseases in much of the world occupy places difficult to access for in situ collection or operate across spaces too large to easily or effectively sample. Satellite based sensors allow for synoptic coverage and the routine collection of data over these sites and situations. Curran et al. [31] outline three underlying premises to justify the use of remotely sensed data in the modeling of vector borne diseases: 1) remotely sensed data can be used to provide information on land cover and by association the habitat of species [32], 2) the spatial distributions of vector-borne diseases are related to the habitat of the vector [33], and 3) if these are true, then remotely sensed data can be used to provide information on the spatial distribution of vector-borne diseases [34]. For this reason, remotely sensed data have been used as descriptors in multiple vector-borne disease modeling research studies (see e.g. [25-27,35-41]). In this study, fifteen publicly available LULC products derived from satellite borne remote sensing instruments were examined to identify which could be used to construct a tsetse fly habitat model.

## Methods

### Data

Fifteen public LULC products (Table 1) available from sources including NASA, International Geosphere-Biosphere Programme (IGBP), The Food and Agriculture Organization of the United Nations (FAO), The Global Environment Monitoring Unit at the University of Maryland (UMd), and the Climate Land Interaction Project (CLIP) located within the Center for Global Change and Earth Observations at Michigan State University were examined. All of the LULC products used in this analysis were originally in or converted to a raster format with a spatial resolution of 1 km or 500 m, and cover the entire country of Kenya. Each LULC data set is unique based on its production methods, classification scheme, temporal acquisition date, and intended use.

**Table 1:** The land use land cover data sets that are publicly available for Kenya.

**Data Set**	**Spatial Resolution**	**Classification Scheme**	**Classes in Kenya**	**Temporal Range**	**Platform**
Africover	1:200,000	Regional FAO LCCS	29	1995	LANDSAT (Bands 4,3,2)
CLIPcover	1 km	Combination of GLC2000 and Africover	43	1995/1999 – 2000	NA
GLC2000	1 km	FAO LCCS	22	1999 – 2000	SPOT 4
IGBP DISCover	1 km	IGBP	16	1992 – 1993	NOAA
UMd GLCC	1 km	UMd modified IGBP	11	1992 – 1993	NOAA
MODIS Type 1	1 km	IGBP	16	Produced Annually 2001 – 2004	MODIS Terra
	500 m	IGBP	17	Produced Annually 2001 – 2005	MODIS Terra & Aqua
MODIS Type 2	1 km	UMd modified IGBP	14	Produced Annually 2001 – 2004	MODIS Terra
	500 m	UMd modified IGBP	14	Produced Annually 2001 – 2005	MODIS Terra & Aqua
MODIS Type 3	1 km	LAI/FPAR	9	Produced Annually 2001 – 2004	MODIS Terra
	500 m	LAI/FPAR	11	Produced Annually 2001 – 2005	MODIS Terra & Aqua
MODIS Type 4	1 km	Net Primary Production	9	Produced Annually 2001 – 2004	MODIS Terra
	500 m	Net Primary Production	9	Produced Annually 2001 – 2005	MODIS Terra & Aqua
MODIS Type 5	1 km	Plant Functional Type	11	Produced Annually 2001 – 2004	MODIS Terra
	500 m	Plant Functional Type	12	Produced Annually 2001 – 2005	MODIS Terra & Aqua

Each type MODIS of product is sub divided into 500 m and 1 km data sets.

The IGBP DISCover land cover product produced by the United States Geological Survey (USGS) Land Cover Working Group in 1995 was created using the Advanced Very High Resolution Radiometer (AVHRR) normalized difference vegetation index (NDVI) 10 day composites from April 1992 to May 1993 [42]. The land cover classes were determined using unsupervised classification on the AVHRR NDVI data on a continental scale [43]. The accuracy of the IGBP DISCover land cover product has been estimated at 66.9 percent for overall area weighted accuracy, and an accuracy range of 40 to 100 percent for individual classes [44].

The Global Land Cover Facility at the UMd produced the UMd Global Land Cover Classification (GLCC) LULC data set utilized the same underlying remotely sensed AVHRR NDVI data as the IGBP DISCover land cover product, but employed a decision tree classification method resulting in a different classification scheme [45]. As explained in Hansen and Reed [42] the major difference between the IGBP DISCover and the UMd GLCC classification schemes is the exclusion of permanent wetlands, cropland/natural vegetation mosaic, and ice/snow by the UMd GLCC product. No formal accuracy assessment has been performed on the UMd GLCC product, though the reported agreement between the UMd GLCC product and the IGBP DISCover is 74 percent [42].

**Figure 3:**
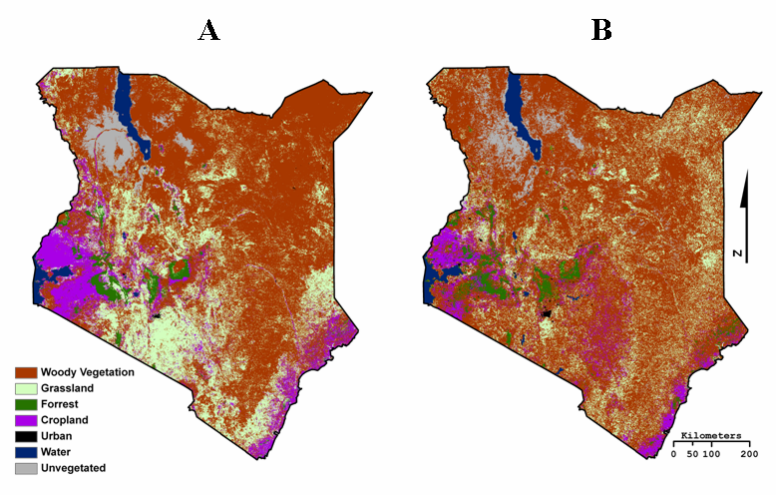
**2001 MODIS Type 1 Global Land Cover 500 m (A) and 1 km (B) spatial resolution**. The classification scheme is simplified to highlight the differences between the two data sets despite the same classification methods. The "Woody Vegetation" class is comprised of mixed forest, shrubland, and savannah land cover, which are considered suitable tsetse fly habitat.

This study also used all 5 types of Moderate Resolution Imaging Spectroradiometer (MODIS) Global Land Cover products in both 500 m and 1 km spatial resolutions (MCD12Q1 & MOD12Q1), which are publicly available from NASA. Although both spatial resolutions of each type of MODIS Global Land Cover product are produced using the same classification method and scheme, the resulting 500 m and 1 km data sets are quite different in the patterns of land cover classes that they display (Figure 3). For this reason, each resolution of each type of MODIS LULC product is considered a separate data set in our analysis.

The MODIS Global Land Cover products were produced annually from 2001 to 2004 for the 1 km data, and 2001 to 2005 for the 500 m data. Only the 2001 data are analyzed here as they are the closest match to the production dates of the validation data. MODIS Type 1 is produced using MODIS NDVI data and the same IGBP global vegetation classification scheme as the IGBP DISCover land cover product [46]. MODIS Type 2 uses the UMd modified IGBP scheme and methodology and the same MODIS NDVI data used to create the MODIS Type 1 land cover product [47]. MODIS Type 3 land cover product is derived from known relationships between estimated leaf area index (LAI) and fraction of photosynthetically active radiation (FPAR) [48]. MODIS Type 4 land cover product is derived from the net primary production (NPP) MODIS products, which measure the growth of the terrestrial vegetation. The MODIS Type 4 classification scheme is primarily geared towards the identification of forest types, such as deciduous broadleaf vegetation and evergreen broadleaf vegetation. MODIS Type 5 land cover product was designed to be used in the Community Land Model for the purposes of climate modeling, and focuses on classifying land cover type based on the plant functional type or plant biome.

Global Land Cover 2000 (GLC2000) was produced by the Joint Research Centre Global Vegetation Monitoring Unit and created over a 14 month period between November of 1999 and December of 2000 using the *VEGETATION *sensor on the SPOT-4 satellite [49]. The classification scheme of GLC2000 used the Land Cover Classification System (LCCS) designed by the FAO [50]. Mayaux et al. [51] have estimated that the overall global accuracy of the GLC2000 product at 68.5 ± 5%. In this study a 26 class African version of GLC2000 was used; 22 of those classes are found within Kenya.

The LCCS was originally developed to aid in the production of the Africover product [52]. Africover was created by combining both computer based unsupervised classification and an expert system supervised classification performed by visual interpretation of mid-1990's era Landsat images by local experts [53]. Several Africover products exist; for this study the spatially aggregated Kenya specific product was used. The original Kenya specific Africover product is in vector format, with 105 LULC classes, and has a nominal spatial scale of 1:200,000. The 105 class Africover vector data set was then converted into a raster data structure with a 1 km spatial resolution, using the highest maximum combined area of all LULC classes found within a grid cell to determine the final raster cell class. To deal with the mixed LULC classes frequently found within the Africover product, the LCCS Code 1 class (i.e. the predominate LULC class in each polygon) was assigned as the overall polygon class. This method reduced the number of LULC classes found in Kenya from 105 to 95, eliminating particular classes not often found or with small surface areas (e.g. snow).

The final LULC data set examined in this study is CLIP Cover produced by the CLIP project at Michigan State University. The CLIP Cover LULC product is a hybrid of GLC2000 and Africover land cover products and essentially uses Africover agricultural data where available and GLC2000 non agricultural land cover data [53]. CLIP Cover was only produced for East Africa.

### Suitability reclassification

Tsetse flies require specific types of land cover generally referred to as woody vegetation [9,13,14,54-56]. However, each subspecies of tsetse fly inhabits distinctive ecosystems with different types of woody land cover. For the purposes of this study the *morsitans *group was selected as the primary focus. The *moristans *group has the greatest spatial distribution in Kenya; with *palpalis *only located along the shore of Lake Victoria and the Ugandan, and *fusca*, whose distributions tend to overlap that of *moristans*, found in isolated pockets of forest and along the Tanzanian border [18,25]. Also, in Kenya, four of the eight tsetse fly species belong to the *moristans *group. Finally, one of the four species within the *moristans *group is *G. pallidipes*, which is considered the tsetse fly species most responsible for transmitting trypanosomiasis in Kenya.

The determination of whether or not a class in a LULC data set contained both the correct type and quantity of woody vegetation suitable for a *moristans *fly was based on the methods outlined in Cecchi et al. [17], which entails examining class descriptions found in the LULC product's metadata or user manuals and comparing it to published habitat requirements (e.g. Table 2). Once land cover classes that contained suitable tsetse fly habitat were identified, the LULC data sets were classified into binary suitable land cover maps.

**Table 2:** MODIS Type 1 LULC classes and their tsetse fly suitability classification.

**Class ID**	**Class Name**	**Class Description**	**Suitable Tsetse** **Land Cover**	**Area of Kenya (km^2^)**
0	Water	Fresh or saline water body	No	12,825
1	Evergreen needleleaf forest	A landscape dominated by trees more than 2 meters tall	Yes	503
2	Evergreen broadleaf forest	A landscape dominated by trees more than 2 meters tall	Yes	15,617
3	Deciduous needleleaf forest	A landscape dominated by trees more than 2 meters tall	Yes	1
4	Deciduous broadleaf forest	A landscape dominated by trees more than 2 meters tall	Yes	899
5	Mixed forests	A landscape dominated by trees more than 2 meters tall	Yes	716
6	Closed shrublands	A landscape dominated by woody vegetation no more than 2 meters tall	Yes	20,998
7	Open shrublands	A landscape dominated by woody vegetation no more than 2 meters tall	Yes	207,803
8	Woody savannas	A mosaic of grass, trees, and shrubs	Yes	42,972
9	Savannas	A mosaic of grass, trees, and shrubs	Yes	122,514
10	Grasslands	Primary vegetation is grass or grass-like plants	No	97,005
11	Permanent wetlands	A permanent mosaic of water, herbaceous, and woody vegetation	Yes	436
12	Croplands	Lands primarily used for agricultural purposes	No	18,536
13	Urban and built-up	Human built environment	No	1,295
14	Cropland/natural vegetation mosaic	A mosaic of cropland, trees, shrubs, and grasslands	No	13,461
16	Barren or sparsely vegetated	Any land surface with little or no vegetation (e.g. sand/rock/salt pans)	No	31,448

**Figure 4:**
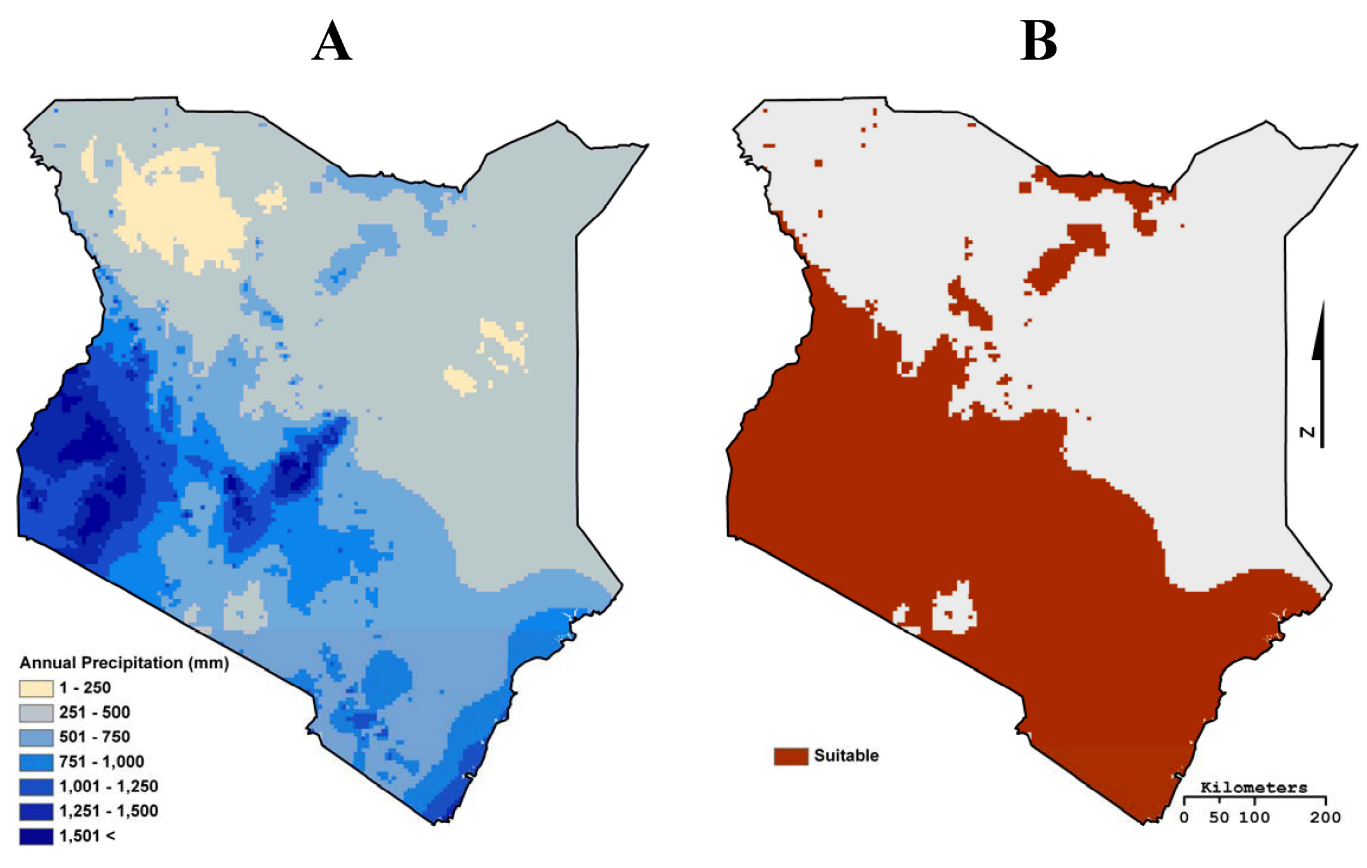
**Map A is a 1 km resolution annual precipitation data set from WorldClim for the year 2000 **[57]. The WorldClim precipitation data set was classified to create Map B, a binary precipitation suitability map.

**Figure 5:**
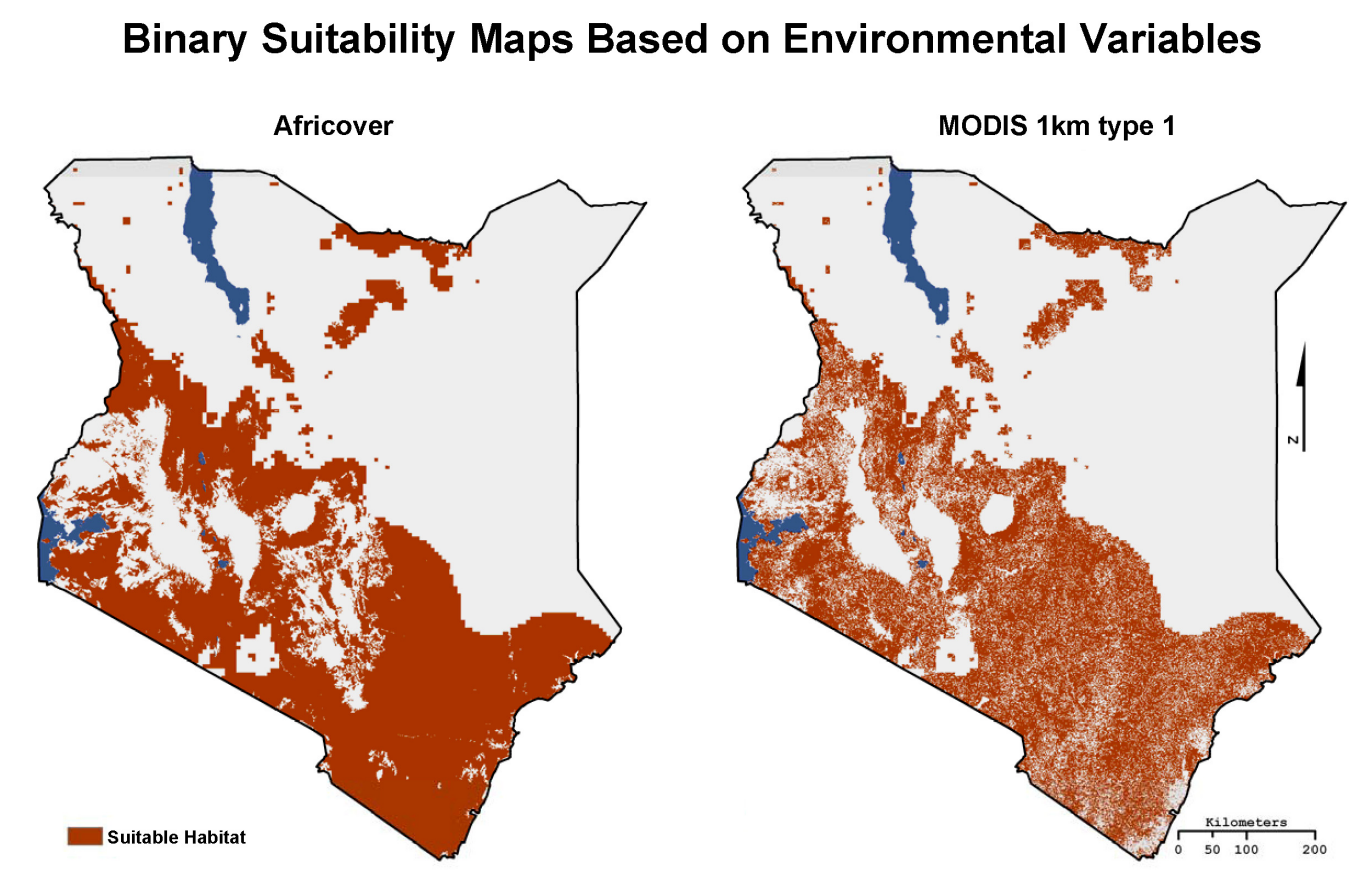
**The binary suitability maps created when the Africover and MODIS 1 km type 1 LULC products were combined with elevation and precipitation data**.

Tsetse flies are also limited by environmental variables such as temperature, humidity, and soil moisture. No in situ country wide humidity or soil moisture data are available, so following Leak [55], we used 500 mm as a proxy for the minimum level of precipitation for tsetse survival and the 1 km resolution annual precipitation data set from WorldClim for the year 2000 [57] (Figure 4). *Moristans *prefer temperatures in the mid 20ºC range [58]; however, tsetse flies will take advantage of micro habitats created by woody vegetation in order to survive temperatures above 32ºC [59]. Thus, maximum temperature was not considered a major limiting variable as long as the proper moisture regimes and land cover were present. Tsetse fly pupa do require a minimum temperature of roughly 16ºC for survival [58]; as in previous research (e.g. Leak [55]), this study used a maximum elevation of 2200 m as a surrogate for minimum temperature. The resulting binary suitability maps based on land cover, elevation, and precipitation suitability were then combined to create an overall suitability map for each of the fifteen LULC data sets (Figure 5).

### Accuracy assessment

The lack of publicly available country wide tsetse fly census data meant that an alternative ground truth data set had to be identified. The first source of ground truth data used in this study was a 1996 tsetse fly belts map produced by the former Kenyan Trypanosomiasis Research Institute (KETRI), now known as Kenya Agricultural Research Institute Trypanosomiasis Research Centre (KARI-TRC) [16,19]. This map represents the most recent field data on tsetse distributions and shows the general location of tsetse fly belts across Kenya (Figure 2).

**Figure 6:**
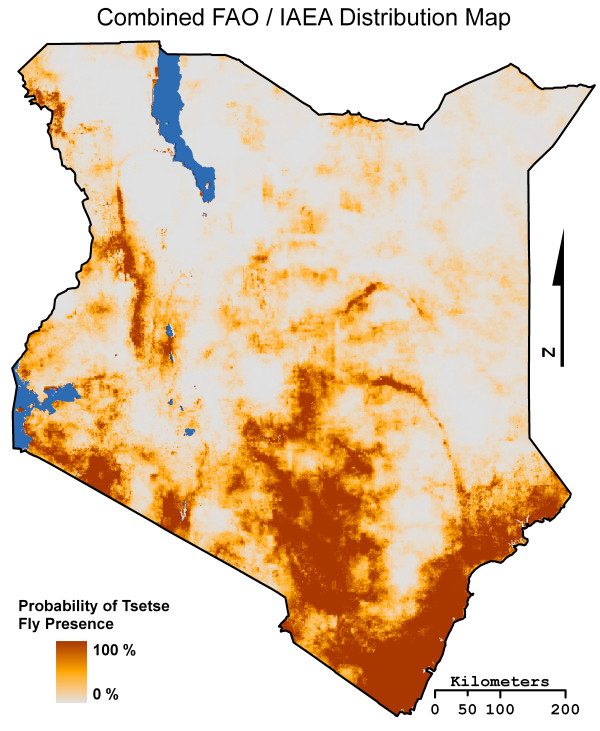
**The Food and Agriculture Organization of the United Nations/International Atomic Energy Agency combined *moristans *tsetse fly species group distribution map**.

A previous study performed by Cecchi et al. [17,60] used the 5 km PAATIS maps as best available tsetse fly distributions data at the continental level. As our study focused on only one country, we chose to use the higher spatial resolution 1 km FAO/IAEA distribution maps as a second source of ground truth data. The FAO/IAEA tsetse fly species distributions maps were produced using logistic regression models, with variables such as NDVI, land surface temperature, infrared reflectance, vapor pressure deficit, air temperature, surface rainfall, elevation, slope, and potential evapotranspiration [27]. The classification scheme of each map displays the predicted percent probability of a particular tsetse fly species being found at any given time. Here, the distribution maps of the four *Glossina *sub-genus species (*austeni*, *morsitans*, *pallidipes*, and *swynnertoni*) were combined to create a *morsitans *group distribution map for all of Kenya. The combined FAO/IAEA *morsitans *distribution map was produced using the mosaic tool in ArcGIS, with maximum mosaic method to ensure the species with the greatest probability would be reported as the pixel probability (Figure 6).

The use of the combined FAO/IAEA *morsitans *distribution map as validation data did pose the problem of using a classification scheme dissimilar to the fifteen LULC binary suitability maps (i.e. percent probability versus binary habitat suitability). Although the classification schemes appear to be different, the variables used to produce the FAO/IAEA distribution maps were ecological suitability variables, and therefore the probability of presence is based on habitat and a direct comparison is possible. In order to account for the differences posed by the percent classification scheme of the combined FAO/IAEA distribution map and the binary LULC suitability maps, an extension of Mapcurves Goodness of Fit (GOF) method of comparison was developed (see Hargrove et al. [61]).

### Mapcurves

**Figure 7:**
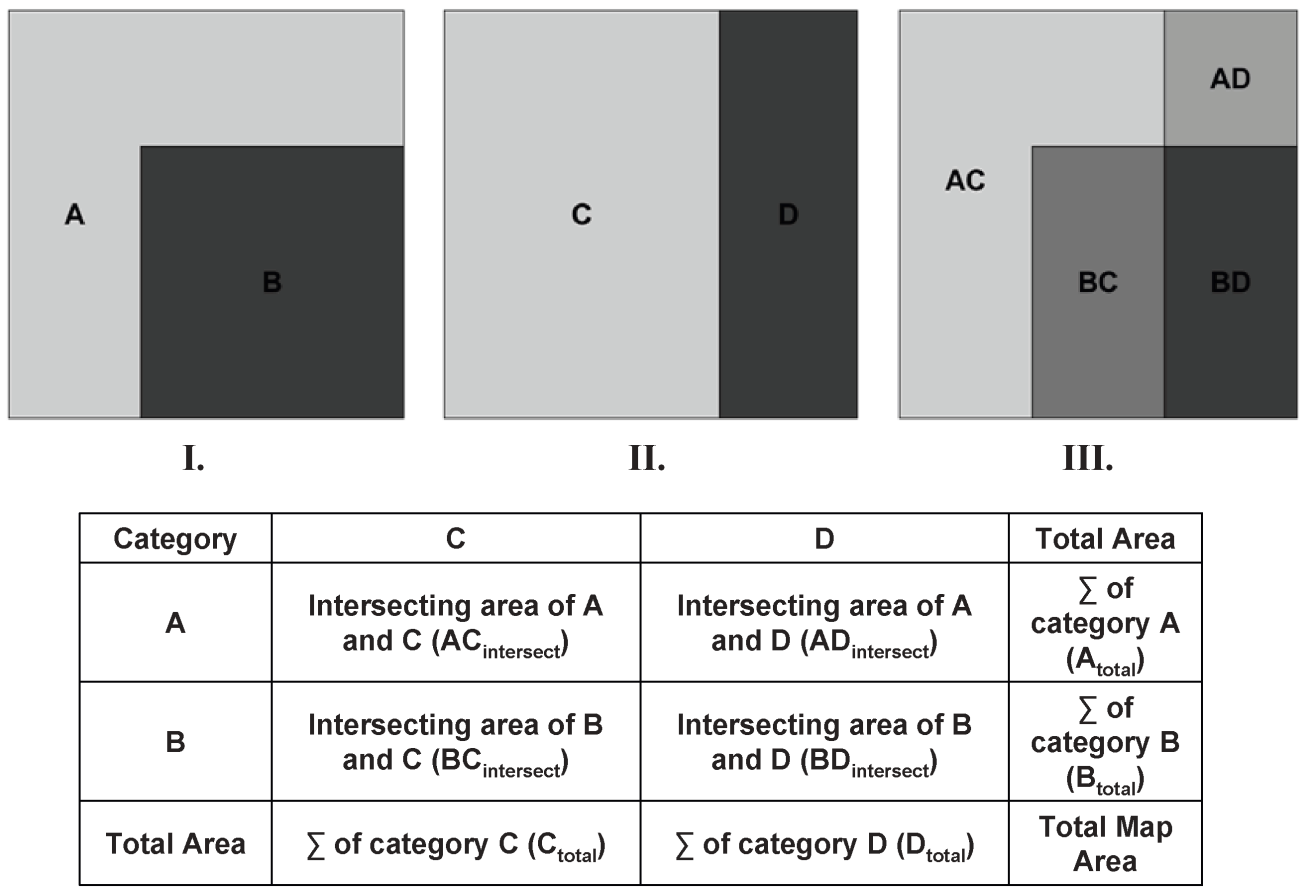
**Example I and II are example categorical data sets to be compared using the Mapcurves GOF approach**. Example III is a visual representation of the cross tabulation matrix created within the GIS environment. The table displayed is a representation of the cross tabulation matrix that would be calculated in the first step of the Mapcurves GOF analysis.

The Mapcurves GOF score is a measure of the degree of spatial autocorrelation between classes of categorical maps with higher Mapcurves GOF scores indicating higher relative positive autocorrelation between classes. The calculation of a Mapcurves GOF score is not limited by differences in resolution, number of classes, or data format, but rather that Map 1 and Map 2 overlap spatially and that the amount of spatial overlap can be measured [61]. One method for calculating the amount of spatial overlap between the classes of two data sets is through the creation of a cross tabulation matrix [62]. The tabulation matrix displays the classes of one data set as rows in the table, and the classes of the other data set as columns [63], and therefore the matrix is comprised of the degree of spatial overlap between the individual classes of the two data sets being compared. Figure 7 shows two example categorical data sets and the cross tabulation matrix that was constructed in the first step of the Mapcurves GOF analysis.

**Figure 8:**
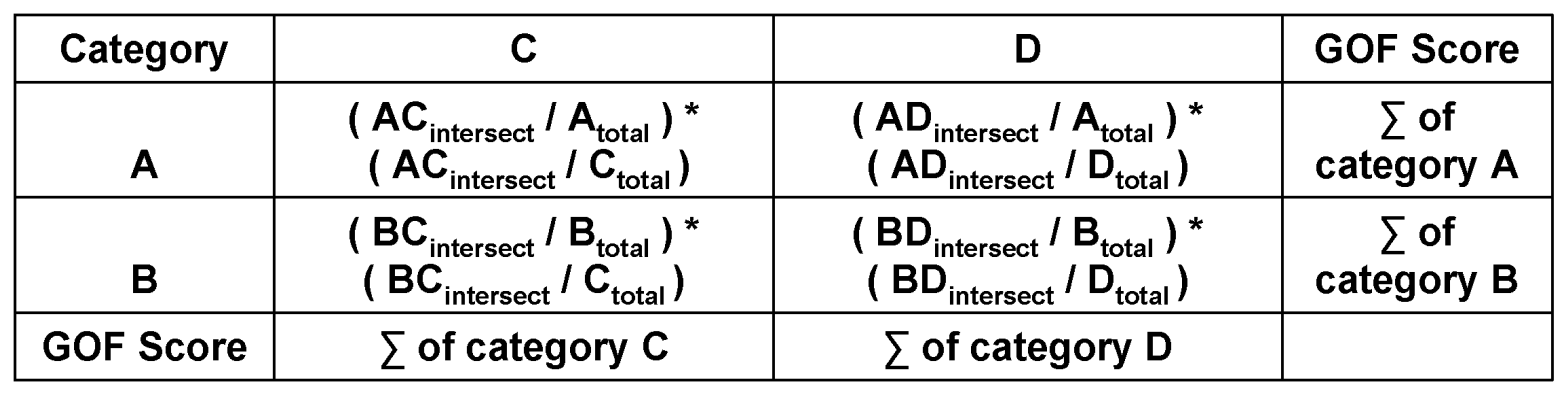
**An example of a weighted ratio comparison matrix for the calculation of a Mapcurves GOF score**.

The resulting cross tabulation matrix table is used to create a weighted ratio comparison matrix. The weighted ratio comparison matrix is constructed by taking the area of two intersecting categories divided by the total area of the Map 1 category, which is then multiplied and weighted by the intersecting area divided by the total area of the Map 2 category. By weighting the proportion of spatial overlap for Map 1 by the proportion of spatial overlap of Map 2, distortion caused by the presence of large, but minimally intersecting categories, is prevented [64]. Each cell within the matrix displays the GOF ratio for the intersecting Map 1 and 2 categories in the associated rows and columns, this information can later be used to determine the best reclassification scheme depending on which map is identified as the reference map. The summing of the rows and columns of the weighted ratio comparison matrix will yield the GOF score of each class category contained in both Map 1 and Map 2 (Figure 8). This information can be used to determine the degree of concordance between categories of the two maps, and is used to create a cumulative ratio frequency distribution.

**Figure 9:**
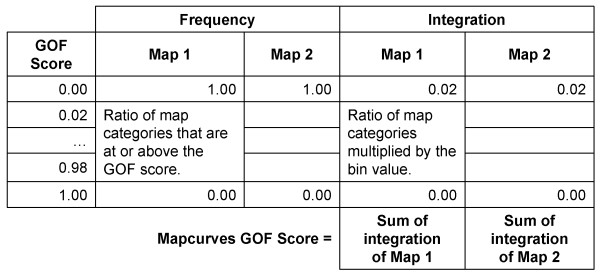
**An example of a cumulative ratio frequency distribution and integration table for the calculation of a Mapcurves GOF score**.

The overall Mapcurves GOF score is the integration of a cumulative ratio frequency distribution (Figure 9). Hargrove et al. [62] used 0.02 as the threshold for the cumulative ratio frequency distribution, and 0.02 was used here as well. The cumulative ratio frequency distribution shows the declining ratio of map categories on the y-axis that still satisfy a GOF Mapcurves score on the x-axis. Once the cumulative ratio frequency distribution has been created, a simple integration will yield the Mapcurves GOF score. This process is then completed for both directions in order to determine which direction has the higher Mapcurves GOF score and therefore the correct direction for reclassification if the reference map has yet to be determined. The direction that yields the highest Mapcurves GOF score is considered to be the best mathematical fit and is considered the reference map. A Mapcurves GOF score of 1.00 represents 100% agreement between the two maps being analyzed (i.e. they are the same map); a low Mapcurves GOF score (e.g. 0.10) is indicative of a high degree of disagreement between the maps.

Once Mapcurves GOF scores have been calculated for both directions, and the reference map has been determined, then the weighted ratio comparison matrix (Figure 8) is used to determine the GOF between individual classes and how to best reclassify the target map based on the classification scheme of the reference map. The reclassification of the target map is implemented by first identifying the highest Mapcurves GOF score in each category's associated row or column in the weighted ratio comparison matrix, then that category is reclassified based on the corresponding class in the reference map.

For this study, the fifteen LULC binary suitability maps were compared to the combined FAO/IAEA distribution map and the 1996 fly belts map using the Mapcurves method. To facilitate the comparison between the FAO/IAEA distribution map and the binary suitability maps, the percent classification scheme of the FAO/IAEA map was classified into a categorical map. A bin value of 0.02 was selected, reclassifying the FAO/IAEA distribution map into a 50 class categorical map (i.e. 2% percent probability per class). A one tail *t*-test, with a significance level of 0.1, was used to detect if any of the LULC data sets had significant levels of agreement with the two ground truth maps.

### Combined suitability map

In an effort to create the best possible LULC data set for modeling tsetse fly in Kenya, we created a hybrid suitability map. This hybrid map was constructed by combining the suitability maps of the five LULC data sets that had the highest level of agreement into a single categorical map. Each class represented the number of LULC data sets that predicted the presence of suitability tsetse fly habitat at that location; for example class 3 means that three LULC data sets predicted suitable tsetse fly habitat.

The combined FAO/IAEA distribution map was then classified to match the same number of classes of our combined suitability map. The classifying of the combined FAO/IAEA distribution map to match our combined suitability map allowed us to run a traditional kappa coefficient and a Mapcurves GOF to test the level of agreement between the two maps. A kappa coefficient is a rescaled proportion of agreement between two data sets, and can be calculated by taking the observed accuracy minus the chance accuracy divided by one minus the chance accuracy as shown below [65,66].



A kappa coefficient of 1.00 indicates perfect agreement between the two maps. The kappa coefficient and the Mapcurves GOF not only test the agreement between the combined FAO/IAEA distribution map and the new combined suitability map, but also explored the level of agreement between the different statistical methods.

## Results

### Binary suitability maps

The fifteen LULC data sets vary widely in the amount of woody vegetation predicted to be in Kenya from roughly 45,000 km^2 ^to 523,000 km^2 ^(Table 3). With the addition of environmental variables and the creation of the binary suitability maps the amount of suitable tsetse fly habitat decreases for each data set and ranges from roughly 31,000 km^2 ^to 205,000 km^2^, still a wide range. The overall decrease in suitable habitat range is primarily caused by low precipitation in the northern parts of Kenya, which creates inhospitable moisture regimes for both tsetse fly adults and pupae.

**Table 3:** The amount of woody vegetation and suitable tsetse fly habitat (when combined with environmental variables) predicted by the LULC binary maps.

**Data Set**		**Amount of Woody Vegetation**		**Predicted Suitability**	
		**Area km^2^**	**% of Kenya**	**Area km^2^**	**% of Kenya**
Africover		515,518	88	205,864	35
CLIPcover		324,896	55	163,340	28
GLC2000		217,938	37	143,683	24
IGBP DISCover		523,527	89	191,849	33
UMd GLCC		280,451	48	149,603	25
MODIS Type 1	1 km	412,459	70	178,669	30
	500 m	364,527	62	126,326	22
MODIS Type 2	1 km	412,403	70	178,647	30
	500 m	387,720	66	146,710	25
MODIS Type 3	1 km	412,319	70	178,626	30
	500 m	387,750	66	146,740	25
MODIS Type 4	1 km	79,768	14	58,741	10
	500 m	45,209	8	31,409	5
MODIS Type 5	1 km	296,386	50	90,609	15
	500 m	311,623	53	80,611	14

The Mapcruves GOF between the 1996 fly belts map and the combined FAO/IAEA distribution map to the LULC binary suitability maps resulted in similar levels of agreement between the fifteen data sets, with a range between 0.52 – 0.59 and 0.53 – 0.65 respectively. When the weighted ratio comparison matrix between each of the binary maps unsuitable class and the 1996 fly belts map and the FAO/IAEA distribution map was examined, high levels of agreement were observed with a range between 0.70 – 0.95 and 0.75 – 0.95 respectively. These observations lead to the conclusion that the high level of agreement between one of the two classes inflated the overall GOF score for each LULC data set, creating a false confidence in the results. For this reason only the GOF between the suitable class of the binary suitability maps and the two maps used as ground truth were examined to determine which data set had the highest level of agreement of tsetse fly presence.

**Figure 10:**
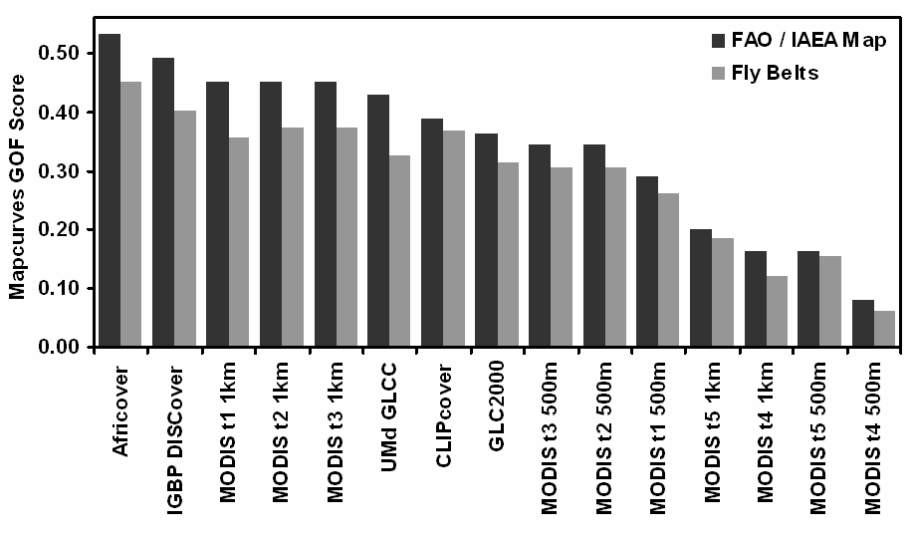
**Mapcurves GOF scores for each LULC data set when compared to the FAO/IAEA combined distribution map**. Data sets are sorted in order from highest to lowest GOF with the FAO/IAEA combined distribution map.

The comparison of the binary suitability maps to the 1996 fly belts resulted in Africover, CLIPcover, IGBP DISCover, and MODIS 1 km Type 1, 2, and 3 products having significant levels of agreement. The comparison of the binary suitability maps to the FAO/IAEA combined distribution map resulted in Africover, IGBP DISCover, UMd GLCC, and MODIS 1 km type 1, 2, and 3 products having significant levels of agreement (Table 4 and Figure 10).

**Table 4:** Results of the Mapcurves GOF analysis between the LULC binary suitable tsetse habitat maps and the combined FAO/IAEA distribution map and the 1996 fly belt map.

**LULC Data Set**		**Mapcurves** **GOF Score**		***t *Score**		***p *Value**	
		**Fly Belts**	**FAO/IAEA**	**Fly Belts**	**FAO/IAEA**	**Fly Belts**	**FAO/IAEA**
Africover		**0.45**	**0.53**	**5.53**	**5.40**	**0.00**	**0.00**
CLIPcover		**0.37**	0.39	**2.71**	1.30	**0.01**	0.11
GLC2000		0.31	0.36	0.78	0.59	0.22	0.28
IGBP DISCover		**0.40**	**0.49**	**3.84**	**4.20**	**0.00**	**0.00**
UMd GLCC		0.33	**0.43**	1.19	**2.44**	0.13	**0.01**
MODIS Type 1	1 km	**0.36**	**0.45**	**2.26**	**3.09**	**0.02**	**0.00**
	500 m	0.26	0.29	-1.04	-1.51	0.63	0.85
MODIS Type 2	1 km	**0.38**	**0.45**	**2.88**	**3.09**	**0.01**	**0.00**
	500 m	0.31	0.34	0.54	0.04	0.30	0.48
MODIS Type 3	1 km	**0.38**	**0.45**	**2.88**	**3.06**	**0.01**	**0.00**
	500 m	0.31	0.34	0.54	0.05	0.30	0.48
MODIS Type 4	1 km	0.12	0.16	-5.86	-5.10	1.00	1.00
	500 m	0.06	0.08	-7.89	-7.45	1.00	1.00
MODIS Type 5	1 km	0.19	0.20	-3.66	-4.05	1.00	1.00
	500 m	0.16	0.16	-4.69	-5.15	1.00	1.00

H_0_: *p *Value ≥ 0.10

H_a_: *p *Value < 0.10

Significant levels of agreement in bold.

### Combined suitability map

**Figure 11:**
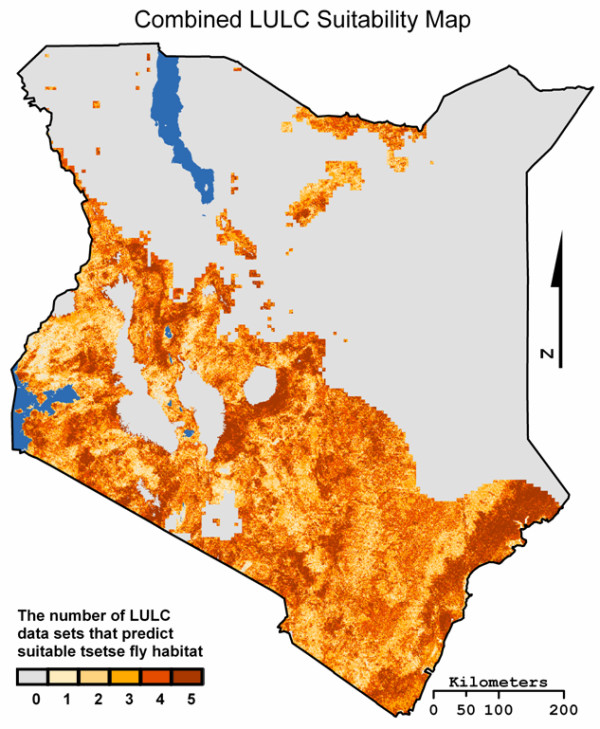
**The suitability map produced when the binary suitability maps for Africover, IGBP DISCover, MODIS t1, UMd Global Land Cover, and GLC2000 were combined**.

Based on the results of Mapcurves GOF analysis of the binary suitability maps the five LULC data sets used to create the combined suitability map were: Africover, IGBP DISCover, MODIS type 1, UMd Global Land Cover, and CLIPcover. MODIS types 2 & 3 were excluded despite their high GOF scores to avoid redundancy given their similarity to MODIS type 1, and alternatively CLIPcover and UMd GLCC were included due to their significant level of agreement with at least one of the two ground truth maps. GLC2000, MODIS 1 km type 4 & 5, and all of the MODIS 500 m LULC data sets were excluded due to their low GOF scores. The resulting suitability map is a categorical map with six classes; 0 representing an area where none of the five LULC data sets predicted suitable tsetse habitat, 5 representing an area where all five of the LULC data sets predicted suitable habitat (Figure 11). The combined FAO/IAEA distribution map was then reclassified into a six class categorical map, from 0 – 100% in ~16.6% increments, to mirror the classification scheme of the newly created combined suitability map.

Examination of the areas of each of the six classes in the FAO/IAEA reclassified and combined LULC maps showed that the largest difference occurred in class 1 where the combined LULC map predicts 14,049 km^2^, and the FAO/IAEA reclassified map predicts 69,147 km^2^, a difference of 55,098 km^2 ^(Table 5). The largest similarity occurs in class 2 with a difference of 5,196 km^2 ^between the two maps.

**Table 5:** The Area of suitable tsetse fly habitat predicted by the combined suitability map and the FAO/IAEA reclassified map.

**Class**	**Combined LULC Suitable Area** **(km^2^)**	**FAO/IAEA Reclassified Suitable Area** **(km^2^)**	**Difference in area between FAO/IAEA and Combined LULC maps** **(km^2^)**
**0**	328,754	376,131	47,377
**1**	13,010	69,147	56,137
**2**	41,578	41,385	193
**3**	80,785	33,531	47,254
**4**	83,197	33,101	50,096
**5**	39,705	33,734	5,971
**Total**	587,029	587,029	207,028

The Mapcurves GOF analysis produced a score of 0.23 (i.e. 23% agreement) between the combined suitability map and the six class FAO/IAEA combined distribution map. The kappa GOF analysis resulted in an observed agreement of 0.55, an expected of 0.39, with a kappa coefficient of 0.26 (Table 6). Both the Mapcurves GOF analysis and the kappa coefficient show that the level of agreement between the two maps is very low.

**Table 6:** The confusion matrix used to calculate kappa coefficient.

		**Combined LULC**							
	**Class**	**0**	**1**	**2**	**3**	**4**	**5**	**Total**	**% Agree**
	**0**	**288,419**	25,126	8,136	3,568	1,734	1,771	328,754	87.7
	**1**	5,614	**2,262**	1,490	1,172	1,218	1,254	13,010	17.4
**FAO/IAEA **	**2**	14,881	7,047	**5,216**	5,054	5,087	4,293	41,578	12.5
**Reclassified**	**3**	28,492	13,206	10,081	**9,563**	9,596	9,847	80,785	11.8
	**4**	28,128	14,531	10,867	9,290	**9,680**	10,701	83,197	11.6
	**5**	10,597	6,975	5,595	4,884	5,786	**5,868**	39,705	14.8
	**Total**	376,131	69,147	41,385	33,531	33,101	33,734	**587,029**	
	**% Agree**	76.7	3.3	12.6	28.5	29.2	17.4		**Kappa = 0.262**

Class agreement between the two data sets is highlighted in bold.

## Discussion

Based on the results of the Mapcurves GOF analysis the top LULC product for use in predicting suitable tsetse fly land cover was Africover. Possible reasons for the Africover product out performing the other LULC products include the higher spatial resolution data used in the creation of the product, local knowledge in the initial classification, and country specific classes. Africover coincidently predicted the largest area of suitable tsetse habitat out of the fifteen LULC products examined. The possibility that this contributed to Africover out performing the other products was explored; however, the apparent relationship between high amount of predicted suitable tsetse fly habitat and high GOF scores does not display a proportional change between percent suitable and GOF scores. If one examines the percent difference of predicted suitability (Table 3) between Africover and IGBP DISCover, a 2% difference is observed, compared to fly belt GOF score difference of 0.05 and FAO/IAEA GOF score difference of 0.04. Similar comparisons between the other LULC data sets GOF scores and percent predicted suitability show no direct proportional relationship, and the general relationship between predicted suitability and GOF scores was considered a negligible result of examining the suitability GOF rather than the overall GOF of each data set.

Although Africover was identified as the top performer, one goal of our analysis was to identify multiple LULC products that can be used to model tsetse fly in Kenya. To that end IGBP DISCover and MODIS type 1, 2, and 3 Global Land Cover at 1 km resolution products were also determined to be strong performers at predicting suitable tsetse fly land cover. The decision on which of the five LULC products to use in the construction of a tsetse fly model can now be made using other factors not directly examined in this study (e.g. accuracy assessments, temporal resolution, data availability). With regards to constructing a model that can predict future tsetse fly distributions based on land use, land cover, climate, and socio economic change, the ability to perform an analysis of LULC change is beneficial. Since currently no plans exist to produce another LULC product similar to Africover, based on the need to model tsetse over time, this product is not considered by us to be the best choice.

An examination of the three MODIS products shows that the GOF scores were nearly identical. To tease out the most favorable product type we examined the results of other LULC data sets that employed the same classification methods and schemes as the MODIS type 1, 2 and 3 products. MODIS type 1 was determined to be the optimal MODIS product since it is based off of the IGBP DISCover classification scheme and method, which had the second highest GOF of all fifteen data sets examined. The similarity between IGBP DISCover and MODIS type 1 allows for a LULC change analysis to be performed since in theory they are directly comparable. In addition to the 2001 data, MODIS produced the 1 km Global Land Cover products annually for 2002, 2003, and 2004. In total, use of the IGBP DISCover and MODIS type 1 products provide five years to construct LULC change trajectories, making them the optimal land cover products to use in modeling tsetse fly.

The results of the combined suitability map constructed from Africover, IGBP DISCover, MODIS t1, UMd Global Land Cover, and CLIPcover showed a notable decrease in the level of agreement compared to the FAO/IAEA map. However, the combined suitability map did allow for a comparison of Mapcurves and Kappa GOF scores, which displayed only a small difference between the two calculated levels of agreement. The similar GOF score calculated by the kappa coefficient and the Mapcurves GOF methods shows that Mapcurves is a viable method of assessing agreement between two maps. Unfortunately the level of agreement produced by both methods is so low that it is clear that that combined suitability map is not an improvement upon the individual binary suitability maps used to create it.

An unexpected result of the GOF analysis was that of the 500 m MODIS LULC products when compared to the 1 km MODIS LULC products. Although the 500 m data has four times the spatial resolution of the 1 km MODIS products, all five of the products were calculated to have insignificant GOF scores. It is our belief that the lower GOF scores are due in part to the over estimation of grassland in southern Kenya. For example, the 500 m type 1 product contains roughly 6% more grassland than the 1 km type 1 product (Figure 3).

The low level of agreement between our maps and the available ground truth data may be partly attributable to the way the ground truth data was constructed. When considering the existing FAO/IAEA products it is important to note that they have not been through peer review, nor do they have a published accuracy statement, thus the low level of agreement may simply be an artifact of accumulative uncertainty. The 1996 fly belts may also have a high degree of uncertainty due to their apparent generalized locations when compared to the more detailed 1973 fly belts produced by Ford & Katondo [7].

Despite the low level of agreement between the binary suitability maps and the combined suitability map when compared to the FAO/IAEA map and the 1996 fly belts, the method we have developed does identify the LULC products that best predict land cover required by tsetse flies. The method we have developed can be used to differentiate between various LULC products and be applied to any such research when there is a known relationship between a species and land cover. The importance of performing this type of analysis can be observed in the results of the GOF scores produced by GLC2000 when compared to Africover. A previous comparison of GLC2000 and Africover performed by Torbick et al. [53] concluded that GLC2000 out performed Africover for predicting natural land cover such as grassland, savannah, and forest. However, in our study we found that the Africover out performs GLC2000 for identifying suitable tsetse fly land cover classes. This discrepancy epitomizes the importance of evaluating the available LULC products and not relying on simple accuracy assessments as each LULC product is different and often has a distinct production method, classification scheme, and intended use.

## Competing interests

The authors declare that they have no competing interests.

## Authors' contributions

The authors contributed equally to the production and approve of the final manuscript.
